# Autoimmune Glial Fibrillary Acidic Protein Astrocytopathy in Children: A Retrospective Analysis of 35 Cases

**DOI:** 10.3389/fimmu.2021.761354

**Published:** 2021-11-22

**Authors:** Hongjun Fang, Wenjing Hu, Zhi Jiang, Haiyan Yang, Hongmei Liao, Liming Yang, Liwen Wu

**Affiliations:** Department of Neurology, Hunan Children’s Hospital, Changsha, China

**Keywords:** autoimmune glial fibrillary acidic protein astrocytopathy, GFAP-IgG antibodies, overlapping autoimmune syndromes, children, GFAP-A

## Abstract

**Objective:**

To analyze the clinical manifestations, imaging, electroencephalography, treatment, and prognosis of 35 cases of autoimmune glial fibrillary acidic protein astrocytopathy (GFAP-A) in children.

**Methods:**

Children hospitalized in the Department of Neurology, Hunan Children’s Hospital, China, between January 2015 and June 2021, owing to autoimmune diseases of the central nervous system were subjected to a cell-based assay (CBA). The assay identified 40 children positive for GFAP-immunoglobulin (Ig)G antibodies in the serum and/or the cerebrospinal fluid. Based on clinical manifestations and imaging characteristics, five children who were only positive for GFAP-IgG antibodies in serum were excluded, and the remaining 35 children were diagnosed with autoimmune GFAP-A. The clinical data derived from the 35 children were retrospectively analyzed.

**Results:**

A total of 35 children, including 23 males and 12 females with a mean age of 6.3 ± 0.6 years, manifested clinical symptoms of fever (62.9%), headache (42.9%), convulsions (42.9%), abnormal mental behavior (51.4%), disorders of consciousness (54.3%), visual disturbance (22.9%), ataxia (11.4%), paralysis (40%), and autonomic dysfunction (25.7%). One child exhibited only the clinical symptom of peripheral facial nerve palsy. Eleven out of 35 children were also positive for other antibodies. In addition to the common overlapping autoimmune syndromes, one case of autoimmune GFAP-A also manifested as Bickerstaff’s brainstem encephalitis. Linear periventricular enhancement upon MRI was significantly less frequent in children (8.5%) than in adults. In pediatric patients, MRI contrast enhancement was principally seen in the meninges and brain lobes. Although repeated relapse (17.1%) and sequelae symptoms (20%) occurred in some cases, most children showed a favorable prognosis. Spearman’s rank correlation showed that the antibody titer was not significantly associated with the severity of the initial disease conditions.

**Conclusions:**

The disease diagnosis in children seropositive for GFAP antibodies only should receive a comprehensive diagnosis based on their clinical symptoms, imaging, electroencephalographic characteristics, and treatment responses. Some patients with relapses should receive repeated gamma globulin and corticosteroid therapy or the addition of immunosuppressants to their therapeutic regimen, and slow-dose tapering of corticosteroids and extended treatment are recommended for patients with overlapping autoimmune syndromes.

## 1 Introduction

Glial fibrillary acidic protein (GFAP) is an intermediate filament protein in astrocytes, with a size between that of microfilaments and microtubules. It is a biomarker for astrocytes, and participates in a variety of biological functions in astrocytes. Through a cell-based assay (CBA) and/or a tissue-based assay (TBA), specific immunoglobulin (Ig)G autoantibodies that selectively target GFAP in astrocytes have been detected in the serum and the cerebrospinal fluid of patients with autoimmune GFAP astrocytopathy (GFAP-A) (1). Autoimmune GFAP-A is an immune (IgG)-mediated inflammation within the central nervous system; its clinical characteristics include signs and symptoms involving the meninges, brain, and spinal cord, including abnormal vision. Although autoimmune GFAP-A has been reported more frequently in adults (especially those over 40 years old) than in children, there are still no standard diagnostic criteria available for this syndrome. Other coexisting neuronal antibodies can also be identified in patients, making disease diagnosis challenging. The specificity of serum GFAP antibodies is still uncertain and requires additional data for further evaluation. A previous study showed the presence of serum GFAP antibodies in patients with Alzheimer’s disease and cancer using ELISA (2). However, it is difficult to determine the significance of positive GFAP antibodies in serum alone in an autoimmune GFAP-A diagnosis. In the present study, we reported the clinical manifestations, characteristics of imaging and electroencephalography (EEG), treatment, and prognostic characteristics of 35 children with autoimmune GFAP-A and positive GFAP antibodies in serum and/or cerebrospinal fluid (CSF). This will provide a reference basis for determining the significance of GFAP antibodies in serum and CSF, and thus allow the formulation of standard diagnostic criteria for autoimmune GFAP-A.

## 2 Materials and Methods

### 2.1 Patient Information and Research Methods

#### 2.1.1 Patient Information

Children hospitalized in the Department of Neurology at Hunan Children’s Hospital (Hunan Province, China) from January 2015 to June 2021 owing to autoimmune diseases of the central nervous system were subjected to CBA to identify 40 children positive for GFAP-IgG antibodies in serum and/or cerebrospinal fluid, followed by collection of their clinical data. Five children only seropositive for GFAP-IgG antibodies were excluded, and the remaining 35 children were diagnosed with autoimmune GFAP-A. The extended stability status scale (EDSS) and the modified Rankin scale (mRS) were used to assess the disease severity in the patients. mRS scores of 4–5 designated severe initial disease, and an mRS score >2 at discharge was considered a poor prognosis for the patient. Clinical tests in this study were approved by the Ethics Committee of Hunan Children’s Hospital, and all patients were informed regarding the clinical testing and provided their consent prior to participating in the study.

#### 2.1.2 All Antibodies Detected in the CSF and Serum

A total of 823 samples of serum (439) and CSF (384) were collected from 499 children that hospitalized in the Department of Neurology, Hunan Children’s Hospital, China, between January 2015 and June 2021, owing to autoimmune diseases of the central nervous system. Of the 823 specimens tested with cell⁃based assays, 50(50/823 [6.08%]) were positive for GFAP-IgG in serum (34/439 [7.74%]) and CSF (16/384 [4.17%]) from 40 patients (patients #1–40 in **Table 1**), including 10 patients both positive for GFAP-IgG in serum and CSF. 33 of the 40 patients had both serum and CSF available, and seven patients had serum only. Serum samples from the 40 patients and CSF samples from 33 patients were used to test other antibodise like MOG, AQP4, NMDAR, AMPA1, AMPA2, LGI1, GABAB, CASPR2, Hu, Yo, Ri, and CV2 antibodies. Antibodies related to peripheral nerve diseases (Gt1a, Gt1b, GQ1b, GM1, GM2, GM3, GD2, and GD3) were tested in seven patients.

**Table 1:** Clinical features, auxiliary examinations, diagnosis and treatment strategies, prognosis in pediatric patients positive for GFAP-IgG.

Patient no. Sex/age of onset	Summary clinical	MRI	EEG	Serum antibody	CSF abnormality	Therapy	Response To therapy	mRS at onset/last	Once lived in ICU
1.M/11.75	vertigo	T2-hyperintense lesions in white matter around posterior horn of bilateral lateral ventricles	normal	GFAP-IgG(1:32)	Wbc:3 P:0.18 Antibody (-)	Prednisone	no change of lesions	1/0	No
2.F/1.67	Fever, dyskinesia	Space occupying lesions in suprasellar region Combined supratentorial hydrocephalus	normal	GFAP-IgG(1:100)	WBC:1 P:0.26 Antibody:NA	Surgery + biopsy	Brain glioma, death	4/5	No
3.M/2.75	epilepsy(Focal attack)	T2-hyperintense lesions in midbrain, cerebral foot, dorsal thalamus, basal ganglia and temporal lobe,	Slow background,left temporal sharp wave	GFAP-IgG(1:100)	WBC:8 P:0.07 Antibody (-)	IVIG,IVMP,AEDS	Brain glioma, aggravated	3/5	No
4.F/6.33	vertigo	T2-hyperintense lesions in bilateral subcortical white matter of frontal, temporal,parietal lobes	NA	GFAP-IgG(1:100)	WBC:2 P:0.11 Antibody (-)	IVIG,IVMP	no change of lesions	2/0	No
5.M/12.41	epilepsy	T2-hyperintense lesions in parasylvian cistern of right parietal lobe, left frontal lobe	right parietal, left frontal sharp wave	GFAP-IgG(1:32)	WBC:3 P:0.412 Antibody (-)	IVIG,IVMP	no change of lesions	2/0	No
6.F/4	psychosis	T2-hyperintense lesions and enhancement in frontal,parietal lobe, thalamus, midbrain	Slow background	GFAP-IgG(1:32)	WBC:8 P:0.25 Antibody (-)	IVIG,IVMP	Symptoms disappeared, Lesion reduced	1/0	No
7.M/11	Limb numbness, Dyskinesia,ataxia	SC:C2-4	Normal	GFAP-IgG(1:32)	WBC:4 P:0.14 Antibody (-)	IVIG,IVMP	Symptoms improved, Lesion reduced	4/1	No
8.M/2.17	epilepsy(Focal attack)	T2-hyperintense lesions in left temporal,occipital lobe	Slow background,left temporal,occipital sharp wave	GFAP-IgG(1:32) after 6 month:GFAP-IgG(1:100)	WBC:55 P:0.13 Antibody (-)	IVIG,IVMP,AEDS	Seizure frequency decreased, cognitive impaired. Lesion reduced	2/0	No
9.M/2	ataxia, Relapse 2 times	T2-hyperintense lesions in white matter around left lateral ventricle	Slow background	GFAP-IgG(1:32)	WBC:32 P:0.19 Antibody :NA	IVIG,IVMP	Retroperitonea-l ganglioma. Lesion reduced	4/1	No
10.M/10.25	Headache,right optic neuritis, Relapse 1 times	T2-hyperintense lesions and enhancement of right optic nerve	normal	GFAP-IgG(1:32)	WBC:0 P:0.25 Antibody (-)	IVMP	Symptoms improved, Lesion reduced	3/0	No
11.M/6	Fever, epilepsy, psychosis, hypersomnia	T2-hyperintense lesions in bilateral thalamus,frontal, temporal, parietal lobes.	Slow background	GFAP-IgG(1:32)	WBC:2 P:0.18 Antibody :NA	IVMP	Symptoms disappeared, Lesion disappeared	3/0	Yes
12.M/10.41	Fever, headache, psychosis, hypersomnia, epilepsy, dyskinesia	T2-hyperintense lesions in parietal lobe, occipital lobe, corpus callosum	Slow background,occipital and posterior temporal electric attack	GFAP-IgG(1:100)	WBC:5 P:0.18 Antibody (-)	IVIG,IVMP,AEDS	Symptoms disappeared, Lesion reduced	5/1	Yes
13.M/8.41	Fever, headache	T2-hyperintense lesions in bilateral subcortical white matter of frontal,parietal lobes	Slow background	GFAP-IgG(1:100)	WBC:1 P:0.13 Antibody :NA	IVMP	Symptoms disappeared, Lesion reduced	2/0	No
14.F/8.08	Headache, psychosis, Relapse 1 times	T2-hyperintense lesions in bilateral thalamus,temporal lobes.	Slow background	GFAP-IgG(1:32)	1.WBC:2 P:0.164 2.WBC:72 P:0.167 Antibody (-)	IVIG,IVMP	Symptoms disappeared, Lesion reduced	2/0	No
15.F/1.92	Fever, headache,hypersomnia	T2-hyperintense lesions in bilateral thalamus, cerebral peduncle, dorsal pons, cerebellar dentate nucleus and left frontal lobe. SC:lesions and mild enhancement in C3-6	Slow background	GFAP-IgG(1:32)	Wbc.231 P.0.47 Antibody :NA	IVIG,IVMP	Symptoms disappeared, Lesion disappeared	2/0	No
16.M/10.58	After mumps,fever, headache,vomit	T2-hyperintense lesions in corpus callosum	Slow background	GFAP-IgG(1:100)	wbc:550 P:0.34 Antibody (-)	IVIG	Symptoms disappeared, Lesion disappeared	1/0	No
17.M/2	Fever, headache, epilepsy, psychosis, hypersomnia, Dyskinesia,Autonomic nerve dysfunction	Demyelinating changes in frontal, parietal, occipital, temporal, cerebellum, brainstem, basal ganglia, thalamus and corpus callosum. Mild enhancement of basal ganglia lesions.Periventricular radial linear enhancement.	Slow background,frontal, central, temporal spike wave	GFAP-IgG(1:10)	Wbc:55 P:0.29 Antibody (-)	IVIG,IVMP	Symptoms improved, Lesion reduced, cognitive, Movement, language impaired.	5/4	Yes
18.F/4.75	right optic neuritis,right limb numbness, Relapse 2 times	Lesions in right optic nerve	Normal	1.MOG (1:100) GFAP( 1:32) 2.MOG (1:320) GFAP( 1:100)	WBC:80 P:0.21 MOG-IgG (1:100)	IVIG,IVMP,Rituximab	Symptoms improved, Lesion disappeared	2/1	No
19.F/5.67	dyskinesia	Enhancement of the anterior root of spinal cord below T 12	Normal	GM1 IgM、GM2 IgM、GFAP-IgG(1:32)	WBC:1 p:0.94 Antibody :NA	IVMP	Symptoms improved, Lesion disappeared	4/3	No
20.M/10.67	Optic neuritis,vertigo	Lesions in bilateral optic nerve, optic chiasm, optic tract	normal	MOG-IgG(1:10)、 GFAP-IgG(1:32)	WBC:6 P:0.24 Antibody (-)	IVMP	Symptoms improved, Lesion disappeared	2/1	No
21、F/9	Fever, headache, hypersomnia, Optic neuritis, dyskinesia,dysuria,Autonomic nerve dysfunction. Relapse 5 times	T2-hyperintense lesions in brainstem,lateral ventricle, bilateral optic nerve, Sc:C3-6,T1-10 Enhancement around lateral ventricle	Slow background	AQP4-IgG(1:32) 、GFAP-IgG(1:32)	WBC:40 P:0.23 AQP4-IgG(1:320)	IVIG,IVMP,Azathioprine,Rituximab	recurrence	5/3	No
22.M/1.58	Fever, hypersomnia, ataxia, dyskinesia, tumor	T2-hyperintense lesions in blateral midbrain, dorsal thalamus, basal ganglia, bilateral cerebral hemispheres, pons and medulla oblongata SC :Cervical spinal cord and below T8	NA	YO IgG、GFAP-IgG(1:100)	WBC:25; p:0.41 Antibody :NA	IVIG,IVMP	Symptoms improved, Lesion disappeared	4/1	No
23.M/13.17	Fever, headache, hypersomnia, seizures, Optic neuritis, left limb dyskinesia,left peripheral facial paralysis,limited abduction of left eyeball, Relapse 1 time	T2-hyperintense lesions in frontal, temporal, parietal, occipital and left optic nerves. strip enhancement in left optic nerves and partial sulcus of bilateral cerebral hemispheres.	Slow background	1.MOG-IgG(1:32) 、 2.GFAP-IgG(1:32) MOG-IgG(1:100)	WBC:360 P:0.2 MOG-IgG(1:32)	IVIG,IVMP+Rituximab	Symptoms improved, Lesion disappeared	5/2	No
24.F/12.25	Fever, psychosis, hypersomnia, seizures,	T2-hyperintense lesions in right temporal, parietal,occipital and thalamus	Slow background	NMDAR-IgG(1:10) 、 GFAP-IgG(1:100)	WBC:37 P:0.15 NAMDR-IgG(1:10)	IVIG,IVMP	Symptoms disappeared, Lesion disappeared	4/1	No
25.M/9.25	psychosis, hypersomnia, seizures,	T2-hyperintense lesions in white matter of right parietal lobe, bilateral basal ganglia, thalamus	NA	NMDAR-IgG(1:10)、GFAP-IgG(1:32)	WBC:120 P:0.21 NMDAR-IgG(1:30)、GFAP(1:32)	IVIG,IVMP	Symptoms improved, Lesion reduced	5/3	No
26.M/11	epilepsy(Focal attack)	T2-hyperintense lesions in right part of cerebral cortex and right optic nerve. Gyrus like enhancement in partial sulcus of right frontal, temporal, parietal and occipital lobes.	Slow background, right temporal Sharp wave	NMDAR-IgG(1:10)、MOG-IgG(1:100)	WBC:28 P:0.21 NMDAR-IgG(1:1)、MOG-IgG(1:100)、GFAP-IgG(1:32)	IVIG,IVMP	Symptoms disappeared, Lesion disappeared	1/0	No
27.F/5.67	headache, psychosis, hypersomnia, visual ghosting	T2-hyperintense lesions in cerebellar parenchyma, brain stem, thalamus, some cerebral hemispheres,right optic nerve. Enhancement of hippocampus and optic nerve. SC:C2-C7、T8-T12.	Slow background	MOG-IgG(1:10)	WBC:26 P:0.15 GFAP-IgG(1:32)、NMDAR-IgG(1:32)	IVIG,IVMP	Symptoms disappeared, Lesion reduced	3/0	No
28.M/3.08	Fever, headache, hypersomnia, weakened knee reflex, ptosis, ataxia	T2-hyperintense lesions and enhancement in bilateral basal ganglia, thalamus and brainstem, bilateral cerebellar hemispheres. Strip enhancement of bilateral temporal, parietal, occipital meninges	Slow background	GFAP-IgG(1:32)、GM1-IGM	WBC:180 P:0.751 GFAP-IgG(1:32)	IVIG,IVMP	Symptoms disappeared, Lesion reduced	4/0	No
29.F/3.58	Fever, headache	T2-hyperintense lesions in Bilateral cerebral hemispheres, around the fourth ventricle, cerebellum, brain stem white matter and sacrococcygeal meninges. SC :Sacrococcygeal meningeal enhancement	Slow background	Antibody (-)	WBC:341 P:0.4 GFAP-IgG(1:32)	IVIG,IVMP	Symptoms disappeared, Lesion reduced	2/1	No
30.M/7	Fever, nausea, vomiting, dizziness	T2-hyperintense lesions in white matter of Bilateral frontal parietal lobe, bilateral basal ganglia, thalamus and corpus callosum	Slow background	GFAP-IgG(1:32)	WBC:18 P:0.45 GFAP-IgG(1:320)	IVIG,IVMP	Symptoms disappeared, Lesion reduced	2/1	Yes
31.M/1.33	Fever, seizures,hypersomnia,dyskinesia	T2-hyperintense lesions in Bilateral Cerebral lobe and cerebellum, brain stem and corpus callosum Meningeal enhancement, periventricular atrophy and right frontal subdural effusion	Slow background,occipital sharp wave	Antibody (-)	WBC:50 P:0.60 GFAP-IgG(1:32)	IVIG,IVMP,AEDS	Symptoms improved, Lesion reduced, cognitive, Movement, impaired.	5/4	Yes
32.M/5.58	Fever,headache,vomiting	Normal	normal	GFAP-IgG(1:32)	WBC:600 P:0.85 GFAP-IgG(1:32)	IVIG	Symptoms disappeared	1/1	No
33.F/7.41	Fever, headache, hypersomnia, psychosis, seizures, dyskinesia,dysuria	T2-hyperintense lesions and enhancement in bilateral frontal,parietal, occipital cortex and white matter, basal ganglia, thalamus and periphery of the fourth ventricle. Meningeal enhancement of basal cistern and diffuse enhancement of meninges. SC:lesion and enhancement in T1-L1,Strip enhancement of spinal meninges	Slow background	GFAP-IgG(1:100)	WBC:240 P:1.56 GFAP-IgG(1:32)	IVIG,IVMP	Symptoms disappeared, Lesion disappeared	5/1	Yes
34.F/4.08	Fever, hypersomnia, psychosis,Involuntary action	T2-hyperintense lesions in Bilateral dorsal thalamus and basal ganglia, bilateral frontal parietal white matter and corpus callosum	NA	Antibody (-)	WBC:55 P:0.88 GFAP-IgG(1:32)	IVIG,IVMP	Symptoms disappeared, Lesion disappeared	4/1	Yes
35.M/1.67	Fever,seizures,dyskinesia, coma,peripheral motor nerve damage	T2-hyperintense lesions in bilateral cerebral cortex, subcortical and thalamus	Slow background	GFAP-IgG(1:10)	WBC:16 P:0.53 GFAP-IgG(1:1)	IVMP	Symptoms improved, Lesion reduced, cognitive, Movement, impaired.	5/4	Yes
36.M/4.25	Fever, headache, hypersomnia, psychosis, hyperhidrosis	T2-hyperintense lesions in bilateral thalamus, frontal and temporal lobes. Leptomeningeal enhancement of cerebrum	Slow background	GFAP-IgG(1:32)	WBC:420 P:1.235 GFAP-IgG(1:32)	IVIG,IVMP	Symptoms disappeared, Lesion disappeared	2/1	No
37.M/7.5	Nephrotic syndrome was diagnosed for 5 years. Fever, psychosis, seizures,coma,dysuria	T2-hyperintense lesions in bilateral cerebral cortex and subcortical white matter, bilateral paraventricular white matter. Linear and arc enhancement of bilateral cerebral sulcus and bilateral forehead	Diffuse low voltage	GFAP-IgG(1:32)	WBC:2 P:0.05 GFAP-IgG(1:32)	IVMP,ADES,Tacrolimus	Symptoms improved, Lesion reduced, cognitive, Movement, impaired,epilepsy	5/4	Yes
38.M/3.75	Fever, psychosis, seizures,coma,urinary retention,optic neuritis,	T2-hyperintense lesions in white matter of bilateral cerebral hemispheres, bilateral basal ganglia, thalamus and fourth ventricle. Periventricular radial linear enhancement. Leptomeningeal enhancement of bilateral occipito parietal lobes.	Slow background,frontal sharp wave	GFAP-IgG(1:32)	WBC:400 P:0.63 GFAP-IgG(1:100)	IVIG,IVMP	Symptoms disappeared, Lesion reduced,	5/3	Yes
39.M/1.17	Right peripheral facial paralysis	T2-hyperintense lesions in white matter around bilateral lateral ventricles	NA	GFAP-IgG(1:1000)	WBC:2 P:0.30 GFAP-IgG(1:100)	IVIG	Symptoms improved, Lesion reduced	2/1	No
40.M/10.75	Fever, psychosis, seizures, coma, dyskinesia, hyponatremia, peripheral nerve injury	T2-hyperintense lesions and enhancement in bilateral basal ganglia, caudate nucleus, thalamus, brainstem and cerebellar dentate nucleus. SC:lesions and enhancement in Spinal cord (mainly cervical and thoracic cord). Strip enhancement of meninges in spine and meninges in basal cistern.	Slow background	Antibody (-)	Wbc:382,P:0.84 GFAP-IgG(1:32)	IVIG,IVMP,PLEX	Symptoms improved, Lesion reduced, cognitive, Movement, impaired.	5/3	Yes

M, male, F, female; N, normal; NA, no available; AED, antiepileptic drug; CSF, cerebrospinal fluid; EEG, electroencephalography; MRI, magnetic resonance imaging; mRS, modified Rankin Scale. IVIG, intravenous immunoglobulin; IVMP, intravenous methylprednisolone; PLEX, Plasma exchange;AQP4, aquaporin-4; MOG, myelin oligodendrocyte glycoprotein; NMDAR, N-methyl-d-aspartate receptor; GM, monosialotetrahexosylgangliosid.

#### 2.1.3 Research Methods

For CBA, serum isolated from the patient blood (at a 1:10 dilution) and 2 ml of CFS were used to incubate HEK293T cells on a glass slide placed in a humidified chamber at room temperature for 1 h. The slides underwent three washes in phosphate-buffered saline (PBS) and further incubation with goat anti-human IgG-fluorescein isothiocyanate fluorescence secondary antibodies at room temperature for 2 h. After being washed three times in PBS, the slides were mounted with glycerol-PBS mounting solution under cover slips before being observed under fluorescence microscopy (Germany LAIKA DMI8).

### 2.2 Statistical Analysis

We employed the SPSS v. 20.0 statistical software package (IBM, Armonk, NY) for statistical analysis in this study. Normally distributed measurement data are presented as means ± standard deviation, and counting data are presented as frequency and composition ratio (%). Spearman’s rank correlation was used to compare two continuous variables. P < 0.05 was considered to be statistically significant.

## 3 Results

### 3.1 Exclusion of the Patients Who Were False-Positive for Autoimmune GFAP-A

Of the 40 patients who were positive for GFAP antibodies, we excluded five who were only seropositive for antiGFAP antibodies (see **Table 1** for details of patients #1–#5). Clinical manifestations of patient #2 included recurrent fever, limb weakness, and poor mental response. This patient was seropositive for anti-GFAP antibodies (1:100) but CSF test was not performed. Magnetic resonance imaging (MRI) of the child’s head revealed a suprasellar space-occupying lesion that was diagnosed as a grade IV glioma by histopathologic examination of the biopsy. This child died 1 year after the diagnosis. Clinical manifestations of patient #3 included recurrent fever and focal seizures originating in the temporal area. This patient was seropositive for anti-GFAP antibodies (1:100) but negative in CSF. The MRI of this patient’s head showed large shadowy patches with abnormal signals in the left midbrain and cerebral peduncle, dorsal thalamus, basal ganglia, and temporal lobe. Immunotherapeutic and antiepileptic treatment were not effective in this patient, who was later diagnosed with glioma through biopsy and exhibited further aggravation of clinical symptoms. The clinical manifestations, imaging results, and EEG changes of patients #1, #2, and #3 were not congruent with autoimmune disorders of the nervous system, and their lesions did not reduce in size after immunotherapy. These patients were not considered to have autoimmune GFAP-A and were excluded from the study. The false-positive detection rate based on GFAP-antibody seropositivity in diagnosing autoimmune GFAP-A was 12.5% (5/40). Among the remaining patients, 19 patients who were only positive for GFAP antibodies in serum (patients #6–24 in **Table 1**) were diagnosed with autoimmune GFAP-A based on their clinical manifestations, imaging characteristics, EEGs, and response to immunotherapy after excluding the possibility of other diseases. Together with the 16 patients who were positive for GFAP antibodies in the CSF (patients #25–40 in **Table 1**), 35 pediatric patients diagnosed with autoimmune GFAP-A were ultimately included.

### 3.2 General Conditions

The age at disease onset of the 35 patients with autoimmune GFAP-A (including 23 boys and 12 girls) ranged from 1 year and 2 months to 13 years and 2 months, with a mean value of 6.3 ± 0.6 years. The common symptoms for these patients included fever, headache, convulsions, abnormal mental behavior, disorders of consciousness, visual disturbance, ataxia, paralysis, and autonomic dysfunction. Of these patients, we noted six cases as positive for GFAP-IgG antibodies only in the CSF, 10 cases as positive for GFAP-IgG in both the CSF and serum, and 19 cases as seropositive for GFAP-IgG only. Moreover, 11 of the 35 children were also positive for other antibodies. Of the two patients in whom tumors were detected, one was diagnosed with autoimmune GFAP-A, and the other was diagnosed with a retroperitoneal paraganglioma 1 year later. One patient was diagnosed with yolk-sac tumors on the right testis 1 year before the onset of autoimmune GFAP-A, which showed an overall tumor incidence of 5.7%.

### 3.3 Clinical Characteristics

We found that 20 of the 35 patients had meningoencephalitis; seven had encephalomyelitis, and one had myelitis. One patient showed three ataxic episodes, with a total disease course of over 1 year; and a mass was eventually found in the retroperitoneum of this patient, who was subsequently diagnosed with ganglioglioma. Another patient with peripheral facial nerve palsy underwent a routine head MRI to reveal T2-hyperintense lesions in white matter around the bilateral lateral ventricles, and we detected GFAP antibodies in serum and CSF of this child. We observed two patients whose only clinical manifestation was focal epilepsy, and a routine head MRI revealed demyelination in their brains. These patients also underwent comprehensive detection for GFAP antibodies in serum and CSF. One returned positive results in both serum and CSF, while the remaining patient was seropositive for GFAP antibodies only. Retesting of GFAP antibodies in serum of the latter patient 6 months later showed significantly increased levels. Three other patients only manifested visual disturbances. Nine of the 35 patients displayed early symptoms, such as runny nose, sneezing, sore throat, or coughing before disease onset; one child was diagnosed with mumps one-half month prior to disease onset; another was diagnosed with infectious mononucleosis one-half month before disease onset. We also noted one patient with Epstein-Barr virus infection 1 week prior to the onset of autoimmune GFAP-A. The primary clinical symptoms of the children with autoimmune GFAP-A included fever (22 cases), headache (15 cases), epileptiform abnormalities and seizures (15 cases), mental and behavioral abnormalities (18 cases), disorders of consciousness (19 cases), visual disturbances (8 cases), ataxia (four cases), paralysis (14 cases), and autonomic neuropathy (nine cases). The duration of fever varied for these patients, with the longest being more than 1 month; and autonomic neuropathy was principally manifested as orthostatic hypotension, flatulence, and urinary retention. Eleven patients were admitted to the intensive care unit owing to illness at the time of admission or during treatment. Six of the 35 patients were hospitalized more than twice as a result of disease recurrence; and one was diagnosed with nephrotic syndrome 5 years before the onset of autoimmune GFAP-A **(** **Table 2**).

**Table 2:** Summary of clinical syndromes and associated conditions in patients with GFAP Astrocytopathy.

Feature	Incidence	Slow wave+epileptiform discharge	6/31 (19.35%)
Males: females	23:12	Neuroimaging	
Mean age	6.3 ± 0.6	Brain	29/35 (82.86%)
Intensive care unit	11/35 (31.43%)	Periventricular radial linear enhancement	3/35 (8.57%)
Early symptoms (runny nose, sore throat, cough, etc.)	9/35 (25.71%)	enhancement of other parts	12/35 (34.29%)
Clinical syndrome		spinal cord	9/35 (25.71%)
Meningoencephalitis	20/35 (57.14%)	optic nerve	7/35 (20%)
Meningeal encephalomyelitis	7/35 (20%)	CSF	
Myelitis	1/35 (2.86%)	Normal leukocyte	12/35 (34.29%)
Main symptoms		Elevated leukocyte	23/35 (65.71%)
Fever	22/35 (62.86%)	Tumor	2/35 (5.71%)
Headache	15/35 (42.86%)	Multiple antibodies positive	11/35 (31.43%)
Seizures	15/35 (42.86%)	Therapy	
psychosis	18/35 (51.43%)	IVMP	7/35 (20%)
Disorder of consciousness	19/35 (54.29%)	IVIG	3/35 (8.57%)
optic neuritis	8/35 ( (22.86%)	IVMP+IVIG	21/35 (60%)
ataxia	4/35 (11.43%)	IVMP, IVIG+Second-line immunomodulatory therapy	4/35 (11.43%)
dyskinesia	14/35 (40.00%)	IVMP,IVIG+PLEX	1/35 (2.86%)
Autonomic nerve dysfunction	9/35 (25.71%)	mRS	
peripheral nerve damage	3/35 (8.57%)	On admission mRS (0-3)	17/35 (48.57%)
EEG findings		On admission mRS (4-5)	18/35 (51.43%)
Normal	5/31 (16.12%)	At discharge mRS (0-2)	26/35 (74.29%)
Slow wave background	19/31 (61.29%)	At discharge mRS (3-5)	9/35 (25.71%)

### 3.4 CSF Characteristics

Of the 35 patients, 10 tested normal for CSF and 25 tested abnormal, the latter primarily exhibiting elevated white blood cells (chiefly monocytes); numbers ranged from tens to hundreds per liter, with the highest number being 600 × 10^6^ cells/L. There were 10 cases with elevated CSF proteins, with the highest level being 1.56 g/L; however, a diminution in the CSF glucose was not apparent. We used the EDSS scores to evaluate the patients’ conditions at admission and Spearman’s rank correlation to assess white blood cells in the CSF, with the parameters showing no significant correlation (P > 0.05).

### 3.5 GFAP Antibodies in Blood and CSF

We measured in one of the 35 patients a CSF titer of 1:1 GFAP antibodies, 12 with 1:32 GFAP antibodies, two with 1:100 GFAP antibodies, and one with 1:320 GFAP antibodies in CSF; and in serum, there were two with 1:10 GFAP antibodies, 20 with 1:32 GFAP antibodies, six with 1:100 GFAP antibodies, and one with 1:1000 GFAP antibodies. In contradistinction to previous studies, the antibody titer in the CSF was not higher than the blood antibody titer in the same patients. Analysis of Spearman’s rank correlation exposed no significant correlation between the GFAP antibody titers in the blood and CSF and the severity of the initial illness (P > 0.05).

### 3.6 Overlapping Positivity With Other Antibodies

Eleven (**Table 1** for details of patients #18–#28) of the 35 patients, including five males and six females, exhibited an overlapping positive signal with other antibodies. One patient was positive for anti-N-methyl-D-aspartate receptor (NMDAR) antibodies and four patients positive for myelin oligodendrocyte glycoprotein (MOG) antibodies (for one of these, the clinical manifestation was only optic nerve involvement, which significantly improved after corticosteroid therapy), One was positive for anti-ganglioside (GM)1-IgM and GM2-IgM antibodies (with a clinical manifestation of peripheral nerve damage, and eventual diagnosis of chronic Guillain-Barre syndrome combined with autoimmune GFAP-A). One was positive for anti-GM1-IgM antibodies (clinical manifestations included fever, headache, poor mental state, ophthalmoplegia, ataxia, and weakened knee reflexes, and the patient was clinically diagnosed with autoimmune GFAP-A combined with Bickerstaff brainstem encephalitis). One was positive for anti-aquaporin (AQP)-4 (clinical manifestations were of brain, spinal cord, and optic nerve involvement and six repeated episodes, and the patient was eventually diagnosed with optic neuromyelitis combined with autoimmune GFAP-A and treated with corticosteroids, gamma globulin, azathioprine, and rituximab). One was positive for anti-Yo antibodies (with symptom onset of abnormal gait; lesions involving the midbrain, thalamus, basal ganglia and cerebral hemisphere, pontobulbar region, and cervical spinal cord; and the patient was diagnosed with testicular yolk-sac tumors 1 year before the onset of autoimmune GFAP-A, although the testicular yolk-sac tumors improved after corticosteroid therapy and gamma globulin therapy). Finally, two patients were positive for both anti-non-allergic and mastocytosis-associated rhinitis (NAMAR) and anti-MOG antibodies. One of these children showed clinical manifestation of focal seizures and elevated white blood cells in the CSF; focal slowing and epileptic waves on EEG; signal changes in the right cortex and white matter; enhancement of the right frontal, temporal, parietal, and occipital lobes; and thickening of the blood vessels upon head MRI. Corticosteroid therapy and gamma globulin therapy showed therapeutic efficacy in this patient. The other child manifested meningoencephalomyelitis and optic neuritis, with signal-foci abnormalities in the brain parenchyma, spinal cord, and optic nerve upon head MRI, as well as long segments of the spinal cord involving C2–C7 and T8–T12. Corticosteroid therapy and gamma globulin therapy showed therapeutic efficacy in this patient.

### 3.7 Imaging Analysis

**Figure 1:**
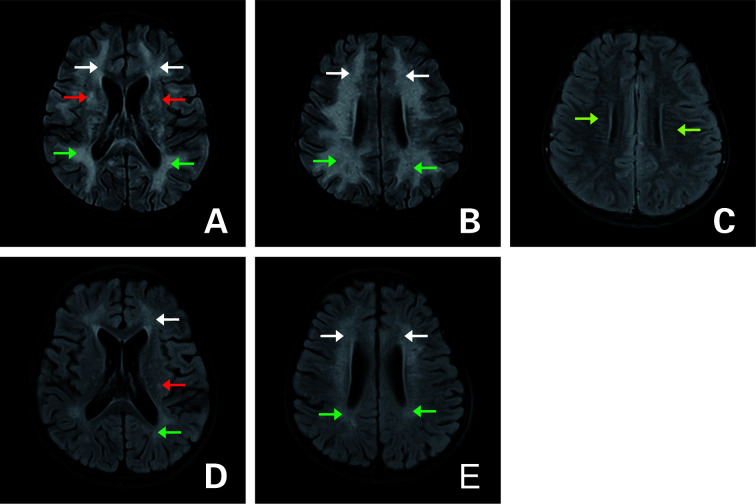
Axial fluid attenuation inversion recovery (FLAIR) MRI of the brain **(A–C)** upon admission and **(D, E)** 3 months later. **(A, B)** T2-hyperintense lesions in the white matter of bilateral cerebral hemispheres (white arrow: frontal lobe; green arrow: parietal lobe), bilateral basal ganglia (red arrow). **(C)** Periventricular radial linear enhancement (light green arrow). **(D, E)** Follow-up images of improved T2 lesions.

**Figure 2:**
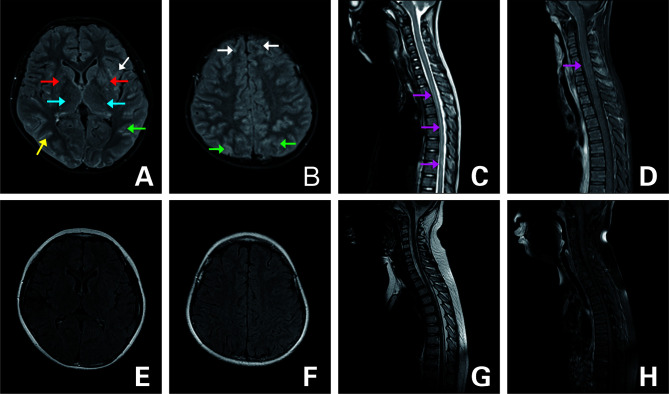
FLAIR MRI of the brain and spine **(A–D)** upon admission and **(E–H)** 3 months later. **(A, B)** T2-hyperintense lesions in bilateral frontal (white arrow), parietal (green arrow), occipital (yellow arrow) cortex and white matter, basal ganglia (red arrow), thalamus (blue arrow), and diffuse enhancement. **(C)** Lesion in the T1-L1 (purple arrow). **(D)** Strip enhancement of spinal meninges (purple arrow). **(E–G)** Follow-up images of improved T2 lesions. **(H)** Follow-up image of the enhancement after it disappeared.

**Figure 3:**
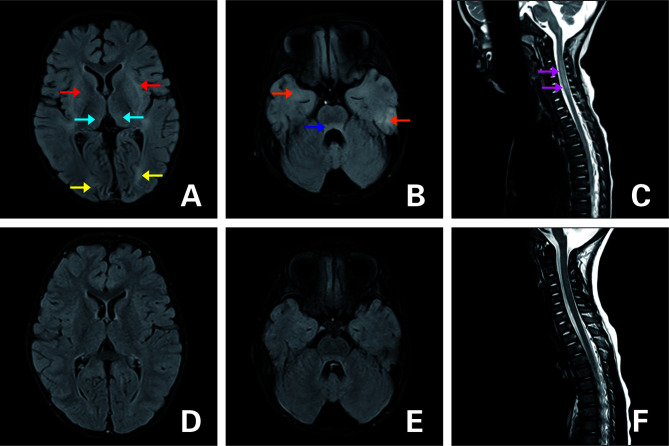
FLAIR MRI of the brain **(A–C)** upon admission and **(D–F)** 3 months later. **(A, B)** T2-hyperintense lesions in the dorsal thalamus (blue arrow), basal ganglia (red arrow), bilateral cerebral hemispheres (yellow arrow:occipital lobe;orange arrow:temporal lobe), and pontine (dark blue arrow). **(C)** Lesions in the cervical spinal cord (purple arrow). **(D–F)** Follow-up images of T2 lesions after they disappeared.

All 35 patients in this study underwent head and spinal MRI. Twenty-two patients showed an abnormal MRI of the head (two of them had optic nerve involvement), two patients only exhibited an abnormal spinal MRI, seven patients had abnormal head and spinal MRIs (two of which had optic nerve involvement); and three patients exhibited an abnormal optic nerve. Lesions upon the head MRI were principally located in the frontal (16 cases), parietal (16 cases), occipital (nine cases), and temporal (nine cases) areas, but were also located in the cerebellum (six cases), brain stem (10 cases), basal ganglia (nine cases), thalamus (17 cases), corpus callosum (seven cases), and around the periventricle (six cases). Of these, 10 patients demonstrated craniocerebral lesions involved in less than three sites; 11 patients had craniocerebral lesions involved in ≥3 and <5 sites; and eight patients showed craniocerebral lesions involved in ≥5 sites. Three patients had an periventricular enhancement on MRI, and 12 patients had an enhancement in other regions, mainly in the meninges, brain lobes, and sulci, which manifested as linear, arc-shaped, extensive, gyriform, and spot-like enhancements. Nine patients displayed an abnormal spinal MRI. We observed two patients with only spinal cord lesions in the cervical region; one with a lesion involving the lumbosacral region; three patients with lesions involving both the cervical and thoracic spinal cord; one patient with lesions involving both the thoracic and lumbar spinal cord; one patient with lesions involving both the thoracic and lumbosacral spinal cord; one patient with lesions involving whole spinal cord; five patients involving >3 spinal cord segments, with the longest involvement in 14 segments; and five patients with spinal cord enhancement, primarily in the meninges and the ventral root of the spinal cord. Imaging results showed that lesions were generally reduced in size and disappeared after treatment. Lesions in 13 patients completely disappeared upon MRI reexamination, with the disappearance time varying from 2 to 5 months after disease onset (**Figures 1**–**3**).

### 3.8 EEG Analysis

Thirty-one patients in our study underwent EEGs: five produced a normal EEG, 19 showed a diffuse slowing of activity across all leads, six showed diffuse slow waves and epileptic waves, and one patient developed diffuse low-voltage activity. Focal seizures developed in two of 31 patients, and one patient displayed focal seizures that originated from the occipital lobe and posterior and temporal areas, with the head MRI showing the lesion in the cortex near the parieto-occipital sulcus and the knees (genu) of the corpus callosum. One patient had multiple focal seizures, two of which originated in the left occipital lobes and three of which originated in the left temporal lobe. The head MRI showed that the lesions were in the left temporal and occipital cortex and white matter. An abnormal region in the EEG was similar to the location of the lesion on the head MRI. Among five patients with normal EEGs, three lesions were noted in the optic nerve, and two only involved the spinal cord on the head MRI.

### 3.9 Therapeutic Analysis

Seven of 35 patients underwent corticosteroid therapy, including three children who underwent gamma globulin monotherapy and 21 cases with gamma globulin plus corticosteroid therapy. Four of the patients (who did not show good therapeutic outcomes after gamma globulin and/or corticosteroid therapy) were also administered immunosuppressants (Rituximab, Azathioprine, and Tacrolimus). One patient who did not show acceptable therapeutic outcomes after gamma globulin and corticosteroid therapy was further subjected to plasmapheresis. Four patients with repeated seizures were administered the antiepileptic drug oxcarbazepine, 10 cases of suspected viral infection in the early stage of admission were treated with the antiviral drug acyclovir, and seven patients experienced paralyzed limbs and intellectual disturbances after their conditions became stable, such that they received supplementation with hyperbaric oxygen therapy and underwent rehabilitation. The average length of the hospital stay of the patients was 25 days. In our study, six of the 35 patients with autoimmune GFAP-A were hospitalized more than twice. Two of our six patients relapsed after receiving gamma globulin and corticosteroid therapy, but their conditions were controlled after supplementation with immunosuppressants. Another patient underwent six repeated hospitalizations within 2 years, with the clinical symptoms relieved after receiving gamma globulin and corticosteroid therapy with the first four hospitalizations. However, the disease recurred in this latter patient several months after the fourth discharge. In addition to the gamma globulin and corticosteroid therapy, this patient was also treated with azathioprine at the fifth hospitalization, but still experienced another recurrence. The patient was ultimately administered rituximab at the sixth hospitalization, and the disease conditions have since stabilized. Three of six patients with a recurrence also experienced overlapping autoimmune syndromes. The remaining three with a recurrence included one with a tumor detected one year after the onset of autoimmune GFAP-A, with the symptoms being relieved after tumor resection; the other two patients with recurrence had their symptoms controlled after another course of corticosteroid therapy subsequent to the second hospitalization.

### 3.10 Prognostic Analysis

At discharge, 26 of the 35 patients had achieved a favorable prognosis, with the remaining nine showing a poor prognosis. All patients received follow-ups *via* telephone or in the outpatient clinic, with an average follow-up duration of 12 ± 5.7 months. At the end of the follow-up period, there were 30 patients who exhibited a good prognosis and five patients with a poor prognosis.

## 4 Discussion

Autoimmune GFAP-A has been reported in five studies, including two from the Mayo Clinic in the United States (3, 4), one from China (5), one from Italy (6), and one from Japan (7). Previous studies have shown that autoimmune GFAP-A occurs at any age but is relatively common in adults, showing no significant difference in the disease incidence between males and females. The median age at disease onset in patients is 44–50 years (8). One report showed that the youngest age at disease onset was three years (4). However, in the present study the youngest age at disease onset was 1 year and 2 months. Females are more than males, which is different from previous studies. The difference may be due to the small sample size of our study.

In these pediatric patients, meningoencephalomyelitis was the primary clinical manifestation of autoimmune GFAP-A; this is consistent with the results in adults in another study (9). However, a small number of patients in our study exhibited other symptoms, including ataxia alone (one case), peripheral facial nerve palsy alone (one case), visual disturbance alone (three cases), and focal epilepsy (two cases). One patient showed obvious cognitive decline after the onset of epilepsy. These patients also displayed abnormal imaging results, and patients with focal epilepsy as the clinical manifestation may also be associated with autoimmune epilepsy. Savaş et al. tested for GFAP antibodies in the blood of 38 children with epilepsy of unknown cause and demonstrated that there were two patients who were seropositive for antiGFAP antibodies (10). In the current study, two patients (5.7%) exhibited focal epilepsy as the only clinical manifestation, showing that GFAP-related autoimmune epilepsy accounted for a specific proportion of children with epilepsy of an unknown cause. Thus, physicians should pay close attention to immunologic indicators in children with epilepsy of an unknown cause. The sole manifestation of peripheral facial nerve palsy in children with autoimmune GFAP-A has not been reported elsewhere in literature, and we were thus the first to address such a case. Therefore, the diverse clinical manifestations of autoimmune GFAP-A in children need to be recognized by physicians.

The specificity of GFAP antibodies in serum remains uncertain and requires more evaluative data. Earlier studies showed that GFAP antibodies were detected in the serum of patients with Alzheimer’s disease, cancers, and brain injury using ELISA (2). However, it is difficult to appreciate the significance of positive-GFAP antibodies in the serum alone in the diagnosis of autoimmune GFAP-A, and it needs to be confirmed in combination with clinical manifestations. We excluded five of 24 patients seropositive for GFAP antibodies because their clinical manifestations and imaging characteristics did not match the diagnostic criteria of autoimmune GFAP-A. Of the five excluded cases, two experienced poor therapeutic outcomes and were eventually diagnosed with glioma by histopathologic examination of biopsy tissue, suggesting that GFAP-antibody seropositivity may also indicate a brain tumor, especially in children with poor therapeutic outcomes and a non-obvious improvement in imaging results. These patients should receive a comprehensive tumor-related examination and undergo biopsy testing if necessary. The diagnosis in patients in which a tumor is found after detecting GFAP antibodies should depend upon clinical symptoms and imaging findings; if these are caused by a tumor space-occupying effect, we propose that “tumor only” should be the diagnosis. However, “autoimmune GFAP-A with a tumor” may be a more appropriate diagnosis if the clinical symptoms and imaging findings suggest paraneoplastic neurologic syndromes. Of the five excluded patients, we noted two with a 1:32 GFAP antibody titer in the blood and three with a 1:100 titer. However, among patients confirmed with autoimmune GFAP-A who carried GFAP antibodies in the blood, there was one patient who displayed a titer of 1:10 GFAP antibodies and 13 who displayed a titer of 1:32 GFAP-antibodies. These findings indicate that the level of GFAP antibodies cannot be used as the sole basis for the diagnosis of autoimmune GFAP-A. The higher GFAP-antibody titer did not enhance the possibility of an autoimmune GFAP-A diagnosis. To diagnose autoimmune GFAP-A in children positive for GFAP antibodies in the blood, it is necessary to pursue a comprehensive analysis that combined clinical manifestations and other auxiliary examinations.

Previous studies showed that GFAP antibody-positivity in CSF achieved high sensitivity and specificity in the diagnosis of autoimmune GFAP-A. In this study, except for four children who were positive for other antibodies and ultimately diagnosed with overlapping autoimmune syndromes in autoimmune GFAP-A, the remaining patients positive for GFAP antibodies in the CSF were diagnosed solely with autoimmune GFAP-A. These findings confirm that GFAP antibodies in CSF reflect high sensitivity in the diagnosis of autoimmune GFAP-A, and this is consistent with the literature (9).

Among patients diagnosed with autoimmune GFAP-A and only seropositive for GFAP antibodies, one manifested recurrent headaches and mild mental and behavioral abnormalities. The first test of this child’s CSF was normal, while the patient’s symptoms were not relieved after conventional treatment; however, a retest of the CSF two weeks later revealed that the number of white blood cells as elevated. Although this patient was seropositive for GFAP antibodies and negative for GFAP antibodies in the CSF, a corticosteroid combined with gamma globulin therapy was administered, and the symptoms were relieved; an ultimate diagnosis of autoimmune GFAP-A was then made in this patient. Another patient with recurrent focal epilepsy who exhibited a 1:32 GFAP antibody titer, lesions in the temporal and occipital lobes on the head MRI, and slow waves in the EEG showed a serum GFAP antibody titer that increased to 1:100 in a retest 6 months later. This patient exhibited cognitive decline 9 months later and underwent immunotherapy, which lowered the frequency of the epileptic episodes and reduced the size of intracranial lesions; autoimmune GFAP-A was ultimately diagnosed. One case in which we made an initial determination of GFAP antibody-positivity in the blood and GFAP antibody-negativity in the CSF had a positive-GFAP antibody reaction in CSF 1 week later, suggesting that children arousing a high degree of clinical suspicion in the diagnosis of autoimmune GFAP-A and with a positive-GFAP antibody test in serum should be subjected to a retest of GFAP antibodies in CSF. However, owing to various reasons, we failed to retest the GFAP antibodies in CSF from all patients who were only seropositive for GFAP antibodies, and we did not verify whether GFAP antibodies first appeared in serum or in CSF. Positivity for GFAP antibodies in serum prior to positive detection in CSF may be due to peripheral nerve injury in patients that occurred earlier than the injury to the central nervous system. For patients positive for GFAP antibodies in serum but not in CSF, a combination of clinical manifestations, imaging, EEG, and response to immunotherapy should be adopted to guide the diagnosis of autoimmune GFAP-A. Among the confirmed cases of autoimmune GFAP-A, GFAP-antibody titers ranged from 1:1 to 1:1,000, which is generally the range for domestic hospitals and testing institutions in China; although the antibody titers in China are typically lower than those reported for other countries (1). We speculate that this may be because we diluted our blood samples 10 times in the test, and different methodologies and evaluation standards used in different countries may also affect antibody titers.

In a previous report from the Mayo Clinic, Flanagan et al. found other autoimmune antibodies in autoimmune GFAP-A, including NMDA, AQP4, and MOG antibodies, which indicate the presence of autoimmune encephalitis and demyelinating disease. Forty-one patients had ≥1 type(s) of antibody (40%) in the blood and CSF, with NMDAR-IgG being the most common type followed by AQP4-Ig (3). Yang et al. demonstrated that 10 of 30 patients positive for GFAP antibodies (33.3%) developed other specific antibodies, with AQP4-IgG being the most common, followed by NMDAR-IgG (11). In our study, 11 of 35 patients positive for GFAP antibodies (31.4%) were positive for other antibodies, showing that overlapping autoimmune syndromes were relatively common in the patients with autoimmune GFAP-A. The overlapping positive signal for MOG antibodies was the most common observation in our patients, followed by NMDA antibodies. Our findings are thus different from those in the aforementioned reports. Of our 11 patients with overlapping autoimmune syndromes, one patient developed autoimmune GFAP-A 1 year after the diagnosis of AQP4 antibody-related neuromyelitis optica; one patient was found to be positive for GFAP antibodies a few months after the diagnosis of MOG-associated disease; and the remaining nine patients showed other autoimmune antibodies contemporaneous with positivity for GFAP antibodies, challenging the diagnosis. There is no clear standard for which autoimmune disorder of the central nervous system, such as demyelinating disease, antibody-related diseases, or even overlapping autoimmune syndromes, is dominant. Although the data from the current study on the 11 cases of overlapping autoimmune syndromes were limited, they nevertheless contributed substantially to the entire database.

Long et al. (5) retrospectively analyzed 19 cases of GFAP-A, conducted brain biopsies in four of the patients, and demonstrated an obvious inflammation in the area around the blood vessels and the presence of T lymphocytes and B lymphocytes in the injured brain tissues, CD3^+^ and CD4^+^ T cells cuffing around brain vessels, accompanied by CD8^+^ T cells CD20^+^ B cells and CD138^+^ B cells. CD138^+^ B cells secrete large amounts of antibodies into the interstitium and areas around the blood vessels, providing an explanation for the majority of studies that showed antibody titers in the CSF to be higher than those in the blood. However, in our study, no significant differences in the GFAP-antibody titers were found between the CSF and blood (P >0.05), which may be related to the small sample size of this study and the analysis time required for determining antibody titers. As an intracellular protein, GFAP cannot be up- or down-regulated on the cell surface, and a similar situation also applies to AQP4 in neuromyelitis optica. Thus, the specific immune mechanisms underlying the generation of GFAP antibodies and the reduction in GFAP expression in astrocytes remain unknown and require urgent clarification in the future.

The present study also revealed abnormalities in the MRIs of 34 patients, displaying lesions involving the meninges, brain, spinal cord, optic nerve, or multiple locations; and 82.9% (29/35) of the patients manifested lesions in brain tissues. These results are in agreement with those in previous reports (89.5% and 75%) (1, 5), and our data showed that brain injury was common in patients with autoimmune GFAP-A. A study from the Mayo Clinic showed that linear periventricular enhancement on MRI was common in patients with autoimmune GFAP-A, affecting 53% of the patients. However, in our study, there were three patients (8.5%) with linear enhancement around the ventricle, and 12 patients (34.3%) with enhancement in other regions, primarily the meninges, brain lobes, and sulci, which manifested as linear, arc-shaped, extensive, gyriform, and spot-like enhancements. The linear periventricular enhancement in the pediatric patients was significantly lower than that in adults, which is consistent with the findings reported by Huang et al. (12). The reasons for the differences in MRI findings between pediatric and adult patients remain unclear and may be due to the lack of pediatric cases overall. In our study, the spinal cord exhibited longitudinally extensive myelitic abnormalities. Usually, myelitic abnormalities present more often in the thoracic and cervical segment, which is largely consistent with previous study findings (5).

Although we noted diffuse slow waves in the EEG tracings from most of our patients (25/35), diffuse slowing of activity in EEGs was not a specific manifestation of autoimmune GFAP-A, as it was also observed in the patients with encephalitis or extensive brain damage. Nevertheless, diffuse, mixed slow waves in EEGs may indicate relatively severe brain damage. Herein, we detected focal seizures in three patients, and the origin of epileptic EEG activity largely matched the region with the MRI lesion. Seven patients with normal EEGs showed lesions in the spinal cord and optic nerve on MRI, while their head MRIs revealed no specific changes, suggesting that there may be a specific correlation among an abnormal head MRI, affected brain regions, and the characteristics of the EEG. Theroux et al. reported a case of autoimmune GFAP-A in a child with an extreme delta brush (EDB) and executed an EEG on the patient; the child was negative for anti-NMDAR antibodies (13). However, we uncovered no EDBs in any of the 35 patients, which may be related to the small sample size and short monitoring time, requiring further follow-up inquiry.

No uniform treatment standard exists for autoimmune GFAP-A. In the current study, most patients with autoimmune GFAP-A received corticosteroids and gamma globulins, which improved clinical symptoms in the majority (85.7%, 30/35). In our study, six of the 35 patients with autoimmune GFAP-A were hospitalized more than twice and had a recurrence rate of 17.1%, which is consistent with the report by Dubey et al. (18%) (4). Although the majority of autoimmune GFAP-A patients responded well to corticosteroid therapy, there remained a certain recurrence rate, suggesting that immunotherapy should not be terminated prematurely. Yang et al. recommended using high-dose corticosteroid therapy to retreat patients with recurrence, although the dose of corticosteroid should then be slowly reduced after at least 3 months of therapy. These authors also recommended using a second-line immunosuppressant if necessary (14). Three of six patients with recurrence who also experienced overlapping autoimmune syndromes in our study showed that the recurrence rate of patients with overlapping autoimmune syndromes (27.3%, 3/11) was greater than that for patients without overlapping autoimmune syndromes (12.5%, 3/24). Ding et al. suggested that the dose reduction in corticosteroids in the children with overlapping autoimmune syndromes should be performed more slowly than that for the children without overlapping autoimmune syndromes, and that second-line immunosuppressants also be used more actively in the recurring cases (15).

In conclusion, the clinical manifestations of autoimmune GFAP-A in children are diverse. In general, pediatric patients with acute or subacute onset experienced clinical manifestations that primarily involved the meninges, brain, spinal cord, and optic nerve; or, they displayed a combination of various symptoms. Head MRIs of the pediatric patients showed periventricular, meningeal, brain lobe, and spinal membrane enhancement. Inflammatory changes in CSF and diffuse EEG waves were possible indicators of disease diagnosis. Corticosteroid therapy in the patients produced good therapeutic outcomes, and assay positivity for GFAP antibodies in CSF displayed high sensitivity and specificity for disease diagnosis in children. Comprehensive analysis was required to confirm the diagnosis of patients who only showed GFAP-antibody positivity in the blood. Given the probability of brain tumors occurring in children with autoimmune GFAP-A, active detection of tumors should be initiated within 2 years of disease onset.

## Data Availability Statement

The original contributions presented in the study are included in the article/supplementary material. Further inquiries can be directed to the corresponding author.

## Ethics Statement

The studies involving human participants were reviewed and approved by the Medical Ethics Committee of Hunan Children’s Hospital. Written informed consent to participate in this study was provided by the participants’ legal guardian/next of kin.

## Author Contributions

HF conducted the literature review and drafted the manuscript. WH, ZJ, HY, HL, and LY made substantial contributions to the conception and interpretation of data. LW was responsible for revising the manuscript critically and has given final approval of the version to be published. All authors contributed to the article and approved the submitted version.

## Funding

This work was supported by grants from the National Natural Science Foundation of China (No. 2021JJ30393).

## Conflict of Interest

The authors declare that the research was conducted in the absence of any commercial or financial relationships that could be construed as a potential conflict of interest.

## Publisher’s Note

All claims expressed in this article are solely those of the authors and do not necessarily represent those of their affiliated organizations, or those of the publisher, the editors and the reviewers. Any product that may be evaluated in this article, or claim that may be made by its manufacturer, is not guaranteed or endorsed by the publisher.
